# Bacterial Diversity in Chinese Rushan Cheese From Different Geographical Origins

**DOI:** 10.3389/fmicb.2018.01920

**Published:** 2018-08-20

**Authors:** Jia Xue, Yi Yang, Zhaoxia Wang, Yurong Guo, Yuyu Shao

**Affiliations:** ^1^College of Food Engineering and Nutritional Science, Shaanxi Normal University, Xi’an, China; ^2^Department of Civil and Environmental Engineering, University of Tennessee, Knoxville, Knoxville, TN, United States

**Keywords:** naturally fermented milk, environmental microorganisms, amplicon sequencing, probiotics, *Lactobacillus*

## Abstract

Rushan cheese, an essential part of the Bai culture, has been produced and consumed for centuries by the Bai people living mostly in Yunnan province of China, however, studies on the naturally occurring microbial communities of Rushan cheese are lacking. In this study, we applied high throughput sequencing technique to analyze the microbial compositions of Rushan cheese samples from three different geographical origins (i.e., Weishan, Eryuan, and Jianchuan). The microbiota in Weishan, Eryuan and Jianchuan Rushan cheese samples were distinct in terms of taxonomic composition and abundance. Linear discriminant analysis (LDA) of effect size (LEfSe) analysis found the characteristic taxonomic species in Weishan Rushan cheese samples were *Lactobacillus pentosus*, *Lactobacillus crustorum*, *Lactobacillus brevis*, *Leuconostoc mesenteroides*, and *Pediococcus pentosaceus*; the representing taxonomic species in Eryuan Rushan cheese samples were *Lactobacillus kefiranofaciens*, *Lactococcus lactis*, *Acetobacter pasteurianus* and *Moraxella osloensis*; by comparison, *Acinetobacter* was enriched in Jianchuan Rushan cheese samples. Characterization of the microbial diversity in Rushan cheese samples from different geographical origins will contribute to the understanding of microorganisms responsible for the Rushan cheese fermentation, and enable us to develop bioresources derived from Rushan cheese in the future.

## Introduction

Dairy products such as milk and yogurt are popular in China; some ethnic minorities like the Bai, Naxi, and Sani, living in the Yunnan province of China, even have historically been involved in making cheese since Ming dynasty (Allen and Allen, 2005). For instance, the Bai people has produced and consumed a unique Rushan cheese for centuries, and the specialized characteristics different from other types of cheeses make Rushan cheese an essential part of the Bai culture (Allen and Allen, 2005). Rushan cheese (known as “milk fan cheese”), thin air-dried strips made from cow milk containing abundant essential nutrients and other substances beneficial to human health, are produced by the procedures: heating, papaya-derived-acid treating, coagulating, squeezing, rubbing, shelling, and then drying (Allen and Allen, 2005). Unlike most of western cheeses’ production requiring the addition of rennet and starter cultures that contain *Lactococcus* and/or *Lactobacillus* (O’Brien et al., 2017), Rushan cheese is directly produced by milk coagulation with acidic solution. During Rushan cheese production, naturally occurring microorganisms from the environment, raw milk, and other sources (e.g., cooking utensils and human hands) contribute to curdling the cheese (Yoon et al., 2016). Evidently, naturally occurring microorganisms play a fundamental and indispensable role in Rushan cheese production.

The importance of microorganisms in producing fermented foods has been well acknowledged; the varieties of cheeses with different textures, aromas, and flavors can be attributed to the diversified functional microorganisms inhabiting the fermented foods (Zheng et al., 2018). Earlier studies relied on traditional methods (e.g., isolation and cultivation) to investigate the microorganisms in the cheese products (Bunesova et al., 2014). However, certain groups of microorganisms are uncultivable, leading to failure of isolation and identification (Shao et al., 2016, 2017b). By combining traditional cultivation-based methods with cultural-independent methods (e.g., PCR-based molecular techniques such as DGGE), the microbial diversity contributing to cheese products has been revealed and cataloged extensively (Ramezani et al., 2017). Compared to traditional cultural-independent methods, metagenomic-based high throughput amplicon sequencing of 16S rRNA genes has the advantages of increased sampling depth, many samples combined in a sequencing run, and cost-effective (Zhang et al., 2017). Amplicon sequencing analysis of 16S rRNA genes were applied to investigate microbial diversities of various types of cheeses from all over the world, such as Austrian artisanal hard cheese (Schornsteiner et al., 2014), Belgian Herve cheese (Delcenserie et al., 2014), Mexican Poro cheese (Aldrete-Tapia et al., 2014), and Azores Pico cheese (Riquelme et al., 2015). However, no studies have been performed to investigate the naturally occurring microbial community in the Rushan cheese and how geographical origins affect microbial structures in the Rushan cheeses that are produced in Yunnan Province.

To fill the knowledge gaps discussed above, this study is aimed at profiling and comparing the microbial communities in Rushan cheese samples collected from Weishan, Eryuan, and Jianchuan of Yunnan province by using 16S rRNA gene amplicon sequencing. The results from this study will help us identify the representative microorganisms in Rushan cheeses, and improve our understanding of microbial-mediated Rushan cheese production processes.

## Materials and Methods

### Preparation of Rushan Cheese

Rushan cheeses from three regions (**Supplementary Figure S1A**), Weishan (W), Eryuan (E), and Jianchuan (J) were made using the same commercial UHT-milk produced by the local Dengchuan cattle, a unique dairy cow species from Dali of Yunnan province, China. The Dengchuan cow milk is rich in fat and protein which is optimal for making local Rushan cheese. In order to guarantee the differences of the cheeses among three regions are just the environments (i.e., the air), the production process of the cheese was highly controlled. The raw milk, acidic/sterilized water, vessels, and bamboo chopstick et al. were the same during production of the cheeses in three regions. In this research, 100 L of the UHT-milk was used to produce Rushan cheese for each region (20 L milk for each batch; five batches in total) at the same day. Rushan cheese samples from Weishan, Eryuan, and Jianchuan were prepared following the same procedures produced by five different local residents in each region. Briefly, acidic water was prepared by boiling papaya (*Chaenomeles sinensis*) and water (1:3, w/w) for half an hour. Then the acidic water was mixed with UHT milk at a volume ratio of 1:2. The mixture was heated to 70°C and stirred until solidification under the influence of heat and acidity. The solidified curd was picked up by bamboo chopsticks, and kneaded in the pot by hand. The homogenized curd was then stretched into an oval slice and wrapped counterclockwise on a bamboo chopstick (**Supplementary Figure S1B**). The curd on the bamboo stick was dried outside for 24 h, and then continued to be naturally dried inside for 2 days. Bamboo stick was removed to obtain Rushan cheese. Fifteen Rushan cheese samples were collected from three regions (numbered W1-W5, E1-E5, and J1-J5), and then transported to the lab in sterilized bags for DNA extraction.

### DNA Extraction and 16S rRNA Gene Amplicon Sequencing

The protein and fat of Rushan cheese were removed before metagenomic DNA extraction according to Escobarzepeda et al. (2016). DNA extraction from the Rushan cheese samples followed the established protocols (Escobarzepeda et al., 2016). The V3 and V4 regions of 16S rRNA gene were amplified using specific primers described in our previous research: universal forward primer 338F (5′-ACTCCTACGGGAGGCAGCA-3′) and the reverse primer 806R (5′-GGACTACHVGGGTWTCTAAT-3′) for PCR (Zhang et al., 2017), done with Phusion^®^ High-Fidelity PCR Master Mix (Thermo Fisher Scientific Inc., Waltham, MA, United States). DNA library was constructed using TruSeq^®^ DNA PCR-Free Sample Preparation Kit (Illumina Inc., San Diego, CA, United States). Amplicon sequencing was then conducted using Illumina HiSeq 2500 platform (Illumina Inc., San Diego, CA, United States).

### Bioinformatics and Statistical Analysis

Bioinformatics analysis of the sequence data has been fully described in our previous research (Shao et al., 2017a; Zhang et al., 2017; Wang and Shao, 2018). Briefly, Qiime pipeline (v1.7.0) was used to filter out low-quality tags. Uparse software (v7.0.1001) was used to cluster effective tags to the OTUs based on 97% similarity of sequences. Representative OTUs with high frequency of occurrence were selected and annotated for taxonomic information (e.g., phylum, family, and genus levels).

Alpha diversity indices (e.g., ACE, chao1, and observed species) were calculated to estimate the species richness and relative diversity level in Rushan cheese samples of different geographical origins (Wang and Shao, 2018). Differences of bacterial community structures among Rushan cheese samples were assessed using a phylogeny-based metric, weighted UniFrac distance. If the calculated weighted UniFrac distance between samples is relatively small, the samples are more similar and share more microbial lineages of common evolutionary history. Principal coordinate analysis (PCoA) was performed using the “ade4” package (Shao et al., 2017a). Linear discriminant analysis (LDA) of effect size (LEfSe) was applied to determine the most discriminant taxa among Rushan cheese samples of different geographical origins; LDA score was set at 2.0. The “anosim” module in “vegan” package of R software was used to conduct Anosim analysis (Wang and Shao, 2018). Taxonomic and phylogenetic tree of microbial flora in Rushan cheese was presented using the GraPhlAn software (Zhang et al., 2017). Analysis of the differences between two groups was done using Wilcoxon rank-sum test. R software was used for plotting and statistical analysis throughout.

## Results

### Sequencing Statistics

The average numbers of pair-end raw reads were 92,564, 88,417, and 87,315 for samples collected from Weishan, Eryuan, and Jianchuan, respectively (**Supplementary Table S1**). After being paired and cleaned, about 52.56, 61.28, and 71.25% of the raw sequences with average lengths 420, 425, and 428 bps for Weishan, Eryuan, and Jianchuan samples were then further clustered for taxonomic assignment (**Supplementary Table S1**).

### Microbial Compositions in Rushan Cheese Samples of Different Geographical Origins

**FIGURE 1:**
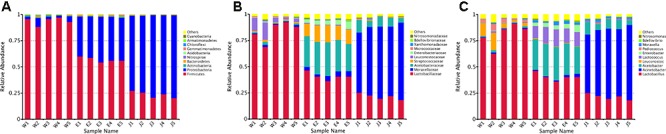
Relative abundance of microbial phylum **(A)**, family **(B)** and genus **(C)** in Rushan cheese samples from Weishan (W), Eryuan (E), and Jianchuan (J).

**FIGURE 2:**
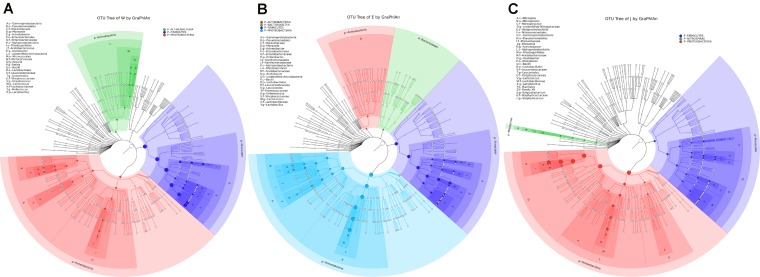
Microbial communities of Weishan **(A)**, Eryuan **(B)** and Jianchuan **(C)** Rushan cheese samples displayed using GraPhlAn showing the most abundant groups at different levels (phylum to genus). The size of the nodes in the cladograms reflected the logarithmically scaled relative abundances of the taxonomic groups. Regions of Weishan, Eryuan, and Jianchuan are indicated by W, E, and J, respectively.

The 255 core OTUs among Rushan cheese samples of different geographical origins were characterized (**Supplementary Figure S2** and **Supplementary Table S2**), and taxonomic levels (i.e., phylum, family, and genus) in each Rushan cheese sample are displayed in **Figures 1**, **2**. The predominant phylum (**Figure 1A**) in Weishan Rushan cheese samples were *Firmicutes*, representing 93.92% of the sequencing reads. Three most abundant families (**Figure 1B**) in Weishan Rushan cheese samples were *Lactobacillaceae* (84.16%), *Leuconostocaceae* (7.79%), and *Acetobacteraceae* (1.40%). The microbiota in Eryuan Rushan cheese samples was dominated by two bacterial phyla, *Firmicutes* (56.98%) and *Proteobacteria* (40.90%); the major families were *Lactobacillaceae* (40.87%), *Acetobacteraceae* (29.64%), *Streptococcaceae* (14.36%), *Enterobacteriaceae* (4.65%), and *Moraxellaceae* (4.20%). While in Jianchuan Rushan cheese samples, the dominant phylum was *Proteobacteria* (75.75%); only 23.54% sequence reads were assigned as *Firmicutes* compared to that in Weishan Rushan cheese samples. Relative abundances of microorganisms at the family level in Jianchuan Rushan cheese samples were 66.34% for *Moraxellaceae*, 21.50% for *Lactobacillaceae*, and 8.06% for *Acetobacteraceae*. Rushan cheeses from different locations had same phyla or families, but relative abundance of them from different locations varied.

**FIGURE 3:**
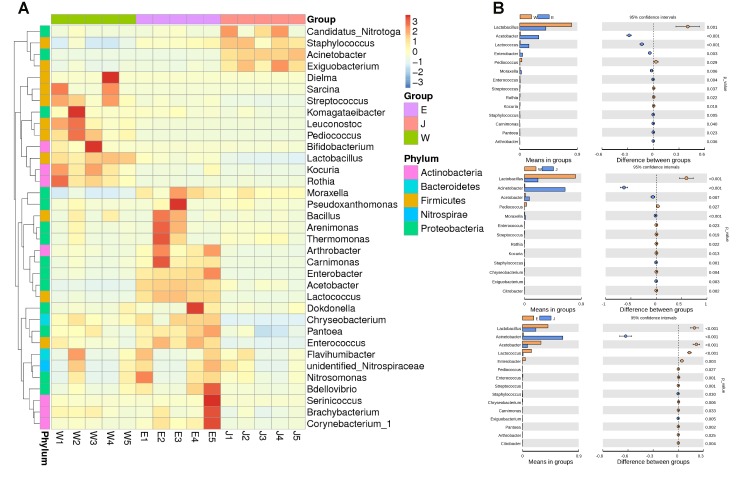
Heatmap of composition and relative abundance of different genera **(A)**, and pairwise comparison of relative abundance of microbial genera in cheese samples from three regions (Weishan-W, Eryuan-E, and Jianchuan-J) based on Wilcoxon rank-sum test **(B)**.

**FIGURE 4:**
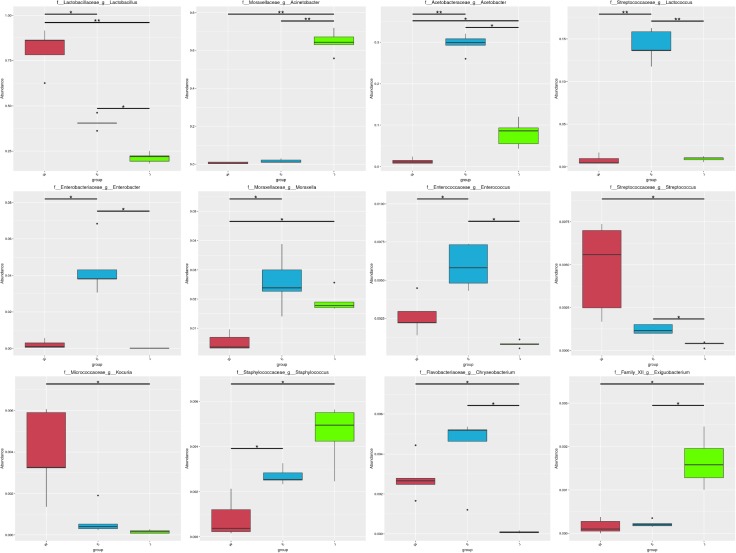
Statistical analysis of genera abundance in Rushan cheese samples of different origins (Weishan-W, Eryuan-E, and Jianchuan-J). ^∗^0.01 < *P* ≤ 0.05; ^∗∗^0.001 < *P* ≤ 0.01.

**FIGURE 5:**
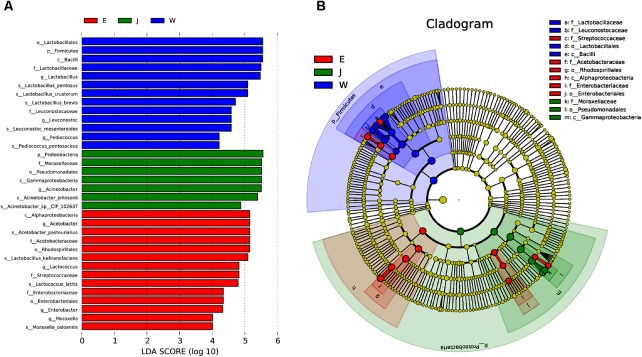
The microbial communities of Rushan cheese samples from Weishan (W), Eryuan (E), and Jianchuan (J) were analyzed using LDA Effect Size (LEfSe) algorithm to determine the optimal characteristic taxa and rank them according to the effect size **(A)**. The cladogram obtained from LEfSe analysis shows the dominant taxa (highlighted by small circles and by shading) in Rushan cheese samples **(B)**.

Microbial structures of all fifteen Rushan cheese samples were further compared at the genus level (**Figures 1C**, **3A**). The dominant genera in Rushan cheese samples from Weishan were *Lactobacillus*, *Leuconostoc*, *Pediococcus*, *Acetobacter*, and *Lactococcus*, representing 80.93, 7.79, 3.22, 1.31, and 0.78% of the microbial community. By comparison, Eryuan Rushan cheeses consisted of *Lactobacillus* (40.73%), *Acetobacter* (29.61%), *Lactococcus* (14.24%), *Enterobacter* (4.37%), and *Moraxella* (2.59%). The most abundant genera in microbial communities of the Jianchuan Rushan cheese samples were *Acinetobacter* (64.41%), followed by *Lactobacillus* (21.44%), *Acetobacter* (7.97%), *Moraxella* (1.92%), and *Lactococcus* (0.94%). Pair-wise microbial comparisons at the genus level are performed in **Figure 3B**, showing the statistically different genera between two groups (*P* < 0.05). Metastat analysis indicated that the relative abundances of 12 genera (e.g., *Lactobacillus*, *Acinetobacter*, *Acetobacter*, *Lactococcus*, *Enterobacter*, *Moraxella*, *Enterococcus*, *Streptococcus*, *Kocuria*, *Staphylococcus*, *Chryseobacterium*, and *Exiguobacterium*) were significant different (*P* < 0.05) among Rushan cheese samples of different geographical origins (**Figure 4**). Moreover, LEfSe analysis revealed five characterizing species in Weishan Rushan cheese samples were *Lactobacillus pentosus*, *Lactobacillus crustorum*, *Lactobacillus brevis*, *Leuconostoc mesenteroides*, and *Pediococcus pentosaceus* (**Figure 5**), while the representing microorganisms in Eryuan Rushan cheese samples were another four species (e.g., *Acetobacter pasteurianus*, *Lactobacillus kefiranofaciens*, *Lactococcus lactis*, and *Moraxella osloensis*). *Acinetobacter* was identified as the representative genus in Jianchuan Rushan cheese samples.

### Microbial Alpha and Beta Diversity in Rushan Cheese Samples

**FIGURE 6:**
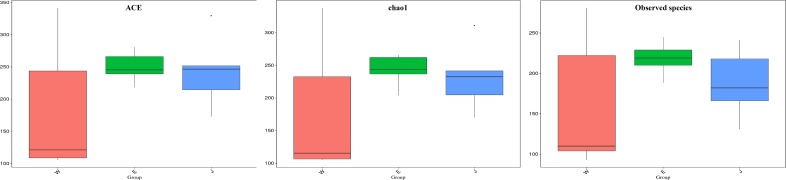
Comparison of microbial alpha diversity indices (ACE, chao1, and observed species) in Rushan cheese samples from three geographical regions (Weishan-W, Eryuan-E, and Jianchuan-J).

**FIGURE 7:**
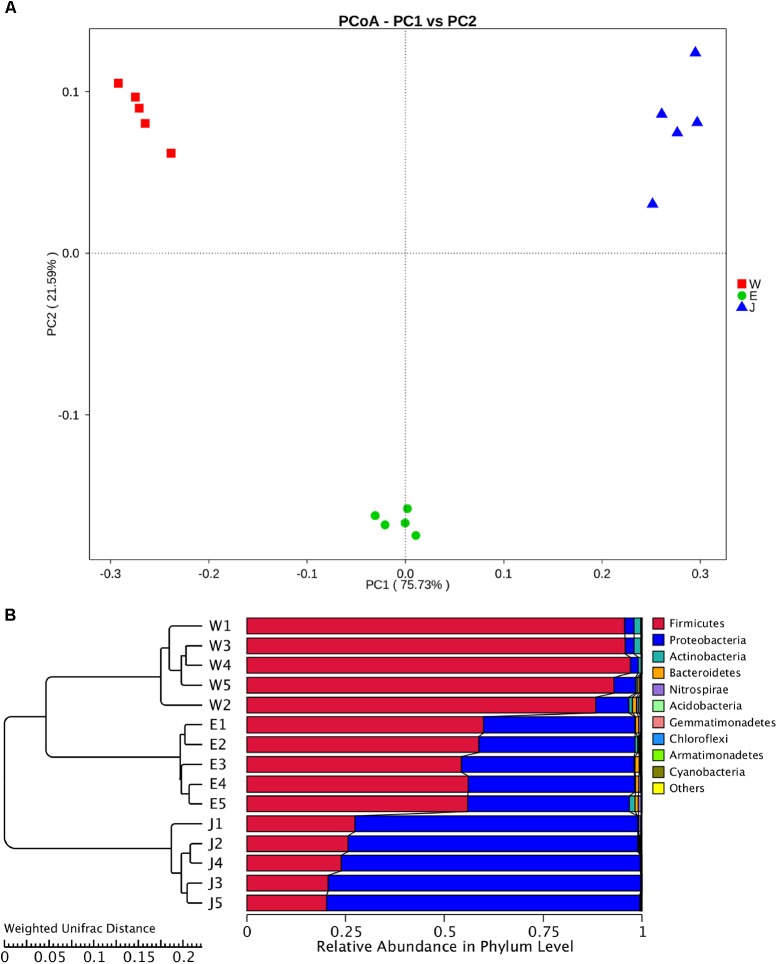
Clustering of microbial community in Rushan cheese samples from three different regions (Weishan-W, Eryuan-E, and Jianchuan-J) based on principal coordinate analysis plot **(A)**. Phylogeny-based metric comparison analysis of dominating phyla in Weishan, Eryuan and Jianchuan Rushan cheese samples **(B)**. Differences of bacterial phyla among Rushan cheese samples were assessed using weighted UniFrac distance.

The highest microbial alpha diversity indices of ACE, chao1, and observed species were detected in Eryuan Rushan cheese samples (**Figure 6**). However, the difference in alpha diversity among three groups of samples was not statistically significant (*P* > 0.05). Weighted UniFrac distances between samples from same locations were relatively small, while different locations resulted in larger differences (**Figure 7**); three groups of microbial communities were significantly different (*P* < 0.001). PCoA revealed strong primary clustering by geographical origins, and the first two components explained about 97% of total variance (**Figure 7**). Microbial composition from the same geographical origin varied significantly less than between geographical origins according to the Anosim analysis (*R* = 1, *P* ≤ 0.011).

## Discussion

Scientific advances in understanding microorganisms have laid the foundation for the industrialization of fermented products manufacture (e.g., milk-derived cheese production). Compared to the industrialized fermented foods, traditional fermented foods are still produced and consumed by native people using locally available raw food materials based on their inherited knowledge and artisanal techniques (Tamang et al., 2016). Rushan cheese is such a traditional fermented food for Bai people in the Yunnan province of China. So far, no scientific investigations have been performed to unravel the microbial communities in the Rushan cheese. In this study, we applied high throughput sequencing technology for the first time to characterize the microbial communities of Rushan cheese samples collected from three different regions in Yunnan province. We found that the microbial communities in Rushan cheese samples of different origins were consisted of different predominant microorganisms. The distinct taxonomic compositions in the Rushan cheese of different geographical origins suggest different microorganisms fulfill the same functions during the Rushan cheese productions, emphasizing the diversity of fermenting microorganisms and the distinction of geographical origins in Rushan cheese production.

High throughput sequencing technique allowed us to investigate the microbial diversities without cultivation, and develop a preliminary understanding of microorganisms’ compositions in the Rushan cheese production. Based on this, we demonstrated that relative abundance of phyla *Firmicutes* in Eryuan Rushan cheese samples was higher than that in Jianchuan, but lower than that in Weishan. In comparison, *Proteobacteria* was higher in Eryuan Rushan cheese samples than that in Weishan, but lower than that in Jianchuan. Interestingly, Eryuan is located between Weishan and Jianchuan, and the distribution of the two predominant phyla presented in the same regular pattern. Further studies are required to investigate the correlation between geographical environments and the microbiota in the cheeses, and potential ways of the microbial acquisition in different regions.

Rushan cheese and Mongolian cheese are produced following the similar procedures involving curdling a mixture of raw milk using the acid solution, in the absence of rennet and starter cultures (Gao et al., 2017). Most of microorganisms were unintentionally introduced into these naturally fermented cheese samples from the environment (e.g., air, cooking utensils hand) during the manufacture (Ceugniez et al., 2016). The microbial communities in Mongolian cheese were dominated by *Lactobacillus* and *Lactococcus* (Gao et al., 2017), like the predominant genera in Eryuan Rushan cheese. These eastern traditional cheeses are different in production technology from the western cheese varieties. Sequencing technique was applied to study the bacterial communities in Pico cheese, revealing its production was a *Lactococcus*-driven process (Riquelme et al., 2015). *Lactococcus* was also the main genus identified in the cheese produced in the area of Herve, Belgium (Delcenserie et al., 2014). However, *Lactobacillus* and *Streptococcus* were found to be the main genera in an artisanal Mexican cheese (Aldrete-Tapia et al., 2014). We found *Lactobacillus* was usually the dominant LAB genus in naturally fermented cheeses, while *Lactococcus*-driven cheese fermentation maybe due to the addition of commercial *Lactococcus*-starter culture. In this study, *Lactobacillus* was the most abundant genus in the Rushan cheese samples compared to the relative low abundances of *Lactococcus*. We found the dominant LAB species in Rushan cheese were *L. pentosus*, *L. crustorum*, *L. brevis* and *L. kefiranofaciens*. Discovery of *Lactobacillus* in Rushan cheese samples suggested the potential benefits of Rushan cheese to the human health and future development of novel functional foods based on Rushan cheese. Another interesting finding was that a dominant genus *Acinetobacter* was identified in Jianchuan Rushan cheese samples. *Acinetobacter* presented in raw milk could increase the viscosity of the milk by producing levan as a capsular polysaccharide, which was reported on curds and soft cheeses surfaces (Gennari et al., 1992).

Environmental bacteria greatly affect the development of cheese characteristics during the manufacture process. In this study, we investigated the microbiota of the artisanal Rushan cheese produced in three regions of Yunnan province of China using high throughput sequencing technique. The results showed differences in bacterial compositions depending on the cheese origins, underlining the effects of geography factor on the final bacterial compositions of Rushan cheese. Our research is important to increase the knowledge about artisanal products and to encourage their production and consumption, and is essential for establishment of the correlation between the environment and the microorganisms to understand how environmental factors affect geographic distribution patterns of microbial communities inhabiting Rushan cheese. It would also be important to characterize the quality of these products, related to the presence of pathogens and spoilage microorganisms to ensure its safety.

## Author Contributions

YG and YS contributed to the experimental design. JX and ZW performed the experiments. YS contributed to the data analysis. YY, YS, and JX wrote the manuscript.

## Conflict of Interest Statement

The authors declare that the research was conducted in the absence of any commercial or financial relationships that could be construed as a potential conflict of interest.

## Supplementary Material

FIGURE S1 Rushan cheese sampling regions **(A)** and picture of Rushan cheese **(B)**.

FIGURE S2 Venn diagram illustrating the number of unique and core OTUs amongst Rushan cheese samples from Weishan (W), Eryuan (E), and Jianchuan (J).

TABLE S1 Sequencing information: summary of raw pair-end reads and QC report. Regions of Weishan, Eryuan, and Jianchuan are indicated by W, E, and J, respectively.

TABLE S2 The 255 core OTUs among Rushan cheese samples of different geographical origins.
