# Supplementary figures and images for: Bacterial Diversity in Chinese Rushan Cheese From Different Geographical Origins
Source: Front Microbiol. 2018 Aug 20;9:1920. doi: 10.3389/fmicb.2018.01920 (PMC6109774; doi:10.3389/fmicb.2018.01920)

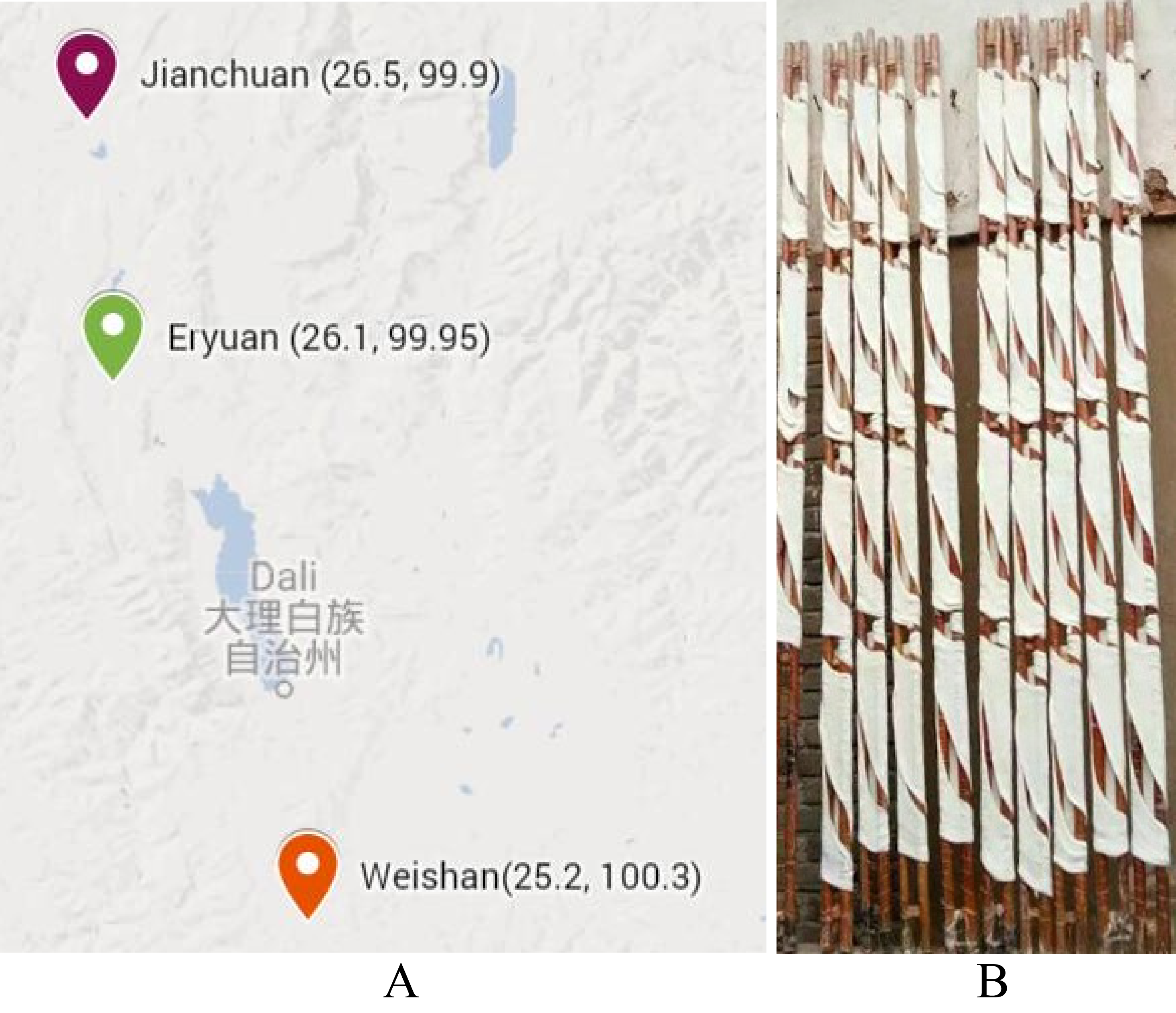

Supplement: FIGURE S1 — Rushan cheese sampling regions (A) and picture of Rushan cheese (B). [file Image_1.TIF]

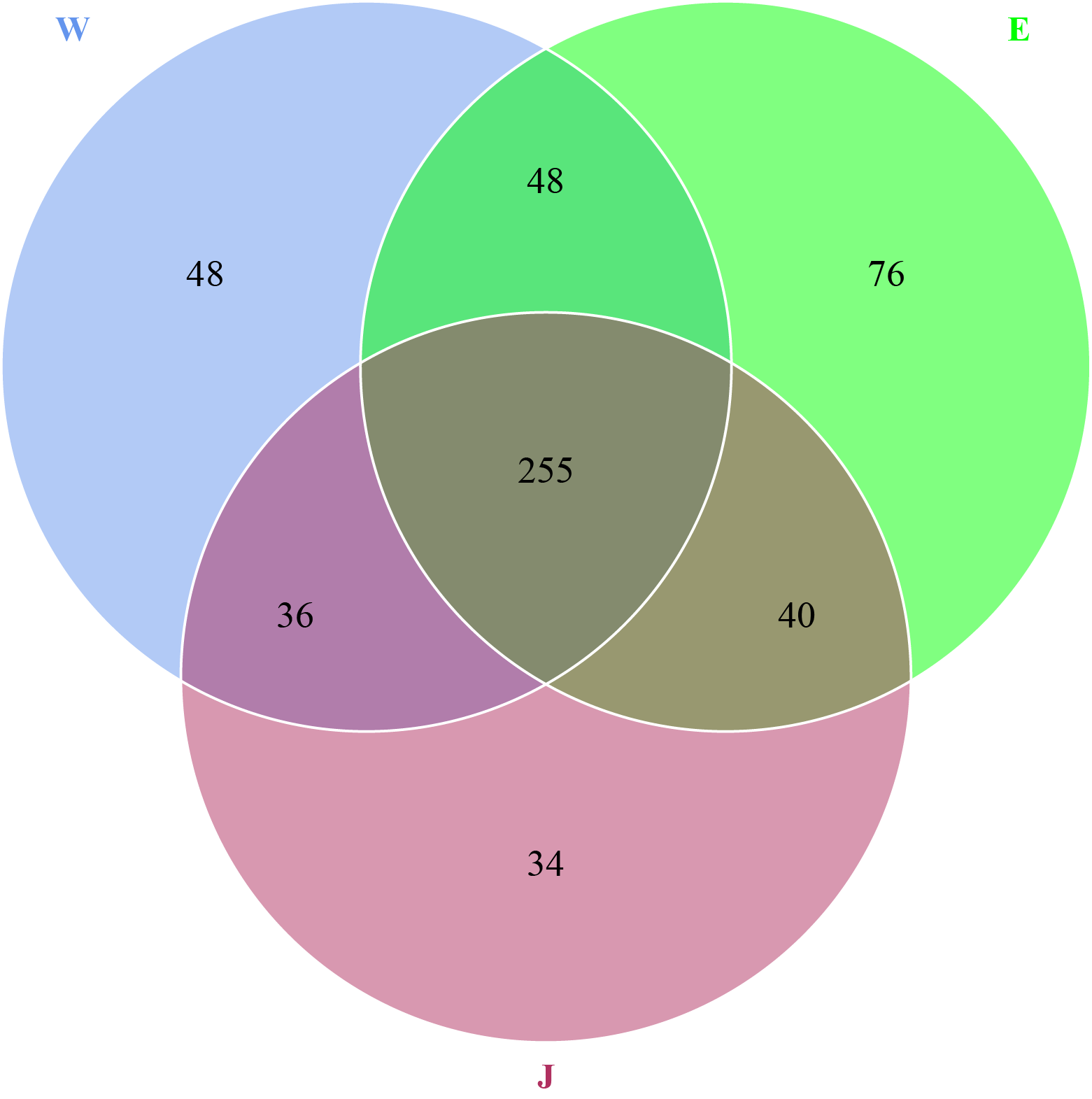

Supplement: FIGURE S2 — Venn diagram illustrating the number of unique and core OTUs amongst Rushan cheese samples from Weishan (W), Eryuan (E), and Jianchuan (J). [file Image_2.TIF]
